# Kinetics and Optimization of Lipophilic Kojic Acid Derivative Synthesis in Polar Aprotic Solvent Using Lipozyme RMIM and Its Rheological Study

**DOI:** 10.3390/molecules23020501

**Published:** 2018-02-24

**Authors:** Nurazwa Ishak, Ahmad Firdaus B. Lajis, Rosfarizan Mohamad, Arbakariya B. Ariff, Mohd Shamzi Mohamed, Murni Halim, Helmi Wasoh

**Affiliations:** 1 Department of Bioprocess Technology, Faculty of Biotechnology and Biomolecular Sciences, Universiti Putra Malaysia, 43400 UPM Serdang, Selangor, Malaysia; nurazwa_ishak@yahoo.com (N.I.); ahmadlajis@hotmail.com (A.F.B.L.); farizan@upm.edu.my (R.M.); arbarif@upm.edu.my (A.B.A.); m_shamzi@upm.edu.my (M.S.M.); murnihalim@upm.edu.my (M.H.); 2 Bioprocessing and Biomanufacturing Research Centre, Faculty of Biotechnology and Biomolecular Sciences, Universiti Putra Malaysia, 43400 UPM Serdang, Selangor, Malaysia

**Keywords:** immobilized lipase, impeller design, response surface methodology, rheology, organic synthesis, kojic acid derivative

## Abstract

The synthesis of kojic acid derivative (KAD) from kojic and palmitic acid (C16:0) in the presence of immobilized lipase from *Rhizomucor miehei* (commercially known as Lipozyme RMIM), was studied using a shake flask system. Kojic acid is a polyfunctional heterocycles that acts as a source of nucleophile in this reaction allowing the formation of a lipophilic KAD. In this study, the source of biocatalyst, Lipozyme RMIM, was derived from the lipase of *Rhizomucor miehei* immobilized on weak anion exchange macro-porous Duolite ES 562 by the adsorption technique. The effects of solvents, enzyme loading, reaction temperature, and substrate molar ratio on the reaction rate were investigated. In one-factor-at-a-time (OFAT) experiments, a high reaction rate (30.6 × 10^−3^ M·min^−1^) of KAD synthesis was recorded using acetone, enzyme loading of 1.25% (*w*/*v*), reaction time of 12 h, temperature of 50 °C and substrate molar ratio of 5:1. Thereafter, a yield of KAD synthesis was optimized via the response surface methodology (RSM) whereby the optimized molar ratio (fatty acid: kojic acid), enzyme loading, reaction temperature and reaction time were 6.74, 1.97% (*w*/*v*), 45.9 °C, and 20 h respectively, giving a high yield of KAD (64.47%). This condition was reevaluated in a 0.5 L stirred tank reactor (STR) where the agitation effects of two impellers; Rushton turbine (RT) and pitch-blade turbine (PBT), were investigated. In the STR, a very high yield of KAD synthesis (84.12%) was achieved using RT at 250 rpm, which was higher than the shake flask, thus indicating better mixing quality in STR. In a rheological study, a pseudoplastic behavior of KAD mixture was proposed for potential application in lotion formulation.

## 1. Introduction

Kojic acid is synthesized by a limited number of microorganisms such as *Aspergillus flavus* and *Aspergillus oryzae*. It is a natural whitening agent and commercially used in certain cosmetic products [1]. The molecular structure of kojic acid comprises a benzene ring with two hydroxyl groups, which makes it polar and slightly soluble in polar organic solvents and water especially at room temperature. However, pure kojic acid is sensitive to moisture, quite unstable, easily oxidized and discolored, thus reducing its quality and price [2,3]. This is because kojic acid in aqueous solution may undergo photo-thermodegradation that changes its chemical structure and biological properties, hence reducing the quality of cosmetic products [2]. Recently, many kojic acid derivatives have been developed with improved stability and solubility in oil and fat in the formulation of cosmetic products [4,5]. Lipophilic derivatives of kojic acid (5-hydroxy-2-hydroxymethyl-4-pyrone) have been synthesized from palm oil or fatty acids by esterification using commercial lipases, including Novozym 435, as biocatalysts [6,7]. Lipase-catalyzed synthesis of ester is challenging due to the poor miscibility of hydrophilic acceptors and hydrophobic acyl donors, resulting in a slow reaction rate [8]. There are various ways to improve the esterification and yield of ester synthesis such as using ionic liquid, super-critical fluid, high speed-homogenization, ultrasound irradiation, modified atmosphere/pressure and molecular sieves [9,10]. Ultrasound-assisted synthesis is an efficient method as it improves the mixing quality and mass transfer rate of the system and, therefore, reduces the reaction time compared to the conventional method [8,9]. High-speed homogenization and high-intensity ultrasound can help to create micro/nano particle sizes and increase the rate of the reaction [11]. The use of supercritical fluid reduces reaction limitation due to heat and mass transfer [11]. However, this equipment is expensive with the use of hazardous chemicals that require extra precautions. Meanwhile, the use of molecular sieves favors synthesis over hydrolysis by attaining low water content in the system [12]. Previous studies have focused on enzymatic synthesis of kojic acid derivatives (KAD) in acetonitrile or solvent-free systems via an esterification process; however, a poor yield of expected derivative products was obtained, which was up to only 30–50% conversion [7]. Moreover, these studies were insufficient since no comparison of solvents for optimization has been carried out and the kinetic study was not well described.

As compared to long chain-like oleic acid, having palmitic acid as an acyl donor to KAD offers certain advantages in cosmetic formulation due to its high penetrability and better depigmenting activity [13]. However, the high melting point introduces a problem in solvent-free systems since palmitic acid can easily solidify at room temperature [7]. Thus, more heat has to be supplied during its synthesis which will add cost due to high energy consumption. In organic solvent systems including acetonitrile, low temperature seemed possible but has been shown to be less efficient for high conversion of KAD [13]. High enzyme loading was required to compensate for the problem during this process, which will also incur more cost. Furthermore, the time of reaction can be reduced if the stability of the enzyme can be maintained at a high temperature. In this study, a suitable solvent for kojic acid derivative synthesis was proposed and compared based on solvent polarity. Among several biocatalysts tested in previous studies, lipase from *Rhizomucor miehei* (Lipozyme RMIM, Novozymes A/S, Bagsværd, Denmark) offered a higher yield of conversion at lower temperature compared to other biocatalysts [4,14]. This sn-1,3-specific lipase was proven to have specificity of reaction, high activity and suitability for catalyzing ester synthesis [13,15]. Hydroxyl group (OH) at carbon 7 of kojic acid was preferably esterified using biocatalysts, namely Novozym 435 (Novozymes A/S, Bagsværd, Denmark), but esterification of OH at carbon 5 of kojic acid possibly depends on the type of lipase and the behavior of the OH group [4,7,15]. Higher yield could be achieved if esterification and modification are made at carbon 5 of kojic acid. Lipozyme RMIM also seems to favor secondary OH of kojic acid to be esterified to fatty acid [4,13].

The selection of biocatalyst is important for a successful biosynthesis manufacturing in large scale biochemical reactors. In general, biocatalyst immobilization often leads to the loss of about 50% of its native activity due to incorrect selection of immobilization method for a specific application [16,17,18,19]. For a long time, the lipase of *Rhizomucor miehei* (RML) has been commercialized in the immobilized form as Lipozyme RMIM where the weak anion exchange resin Duolite ES 562 based on phenol-formaldehyde copolymers has been used as support [14]. Lipases are called interfacial enzymes [20] because they are able to be adsorbed on hydrophobic surfaces of drops of oils, their natural substrates, thanks to the existence of two conformational forms. The closed form is usually the active center isolated from the medium (lipase B from *Candida antarctica* is one of the exceptions [21]) by a polypeptide chain called lid. This lid has a hydrophobic internal face that interacts with the hydrophobic surroundings of the active center [20]. The open form has this lid shifted and exposes a large hydrophobic pocket to the medium. This structure is unstable in aqueous homogenous media but can be strongly adsorbed on the hydrophobic surface of the drops of substrates (oils). This causes the affinity of lipases for hydrophobic surfaces of substrate drops [22]. Lipozyme RMIM possesses a very high activity and good stability even at very low water activity such as anhydrous organic solvents [17]. Thus, it is a good candidate to catalyze the synthesis of KAD via direct esterification (using free fatty acids) where the lower the water activity in the system, the higher the synthetic yields. Lipozyme RMIM is protected from interactions with any external interfaces and may no longer suffer interfacial activation by external interfaces [17,23]. Moreover, the enzyme stability has significantly improved, mainly in the presence of organic solvents, which might be due to the highly hydrophilic microenvironment of the support [14,17,19,23].

**Figure 1:**
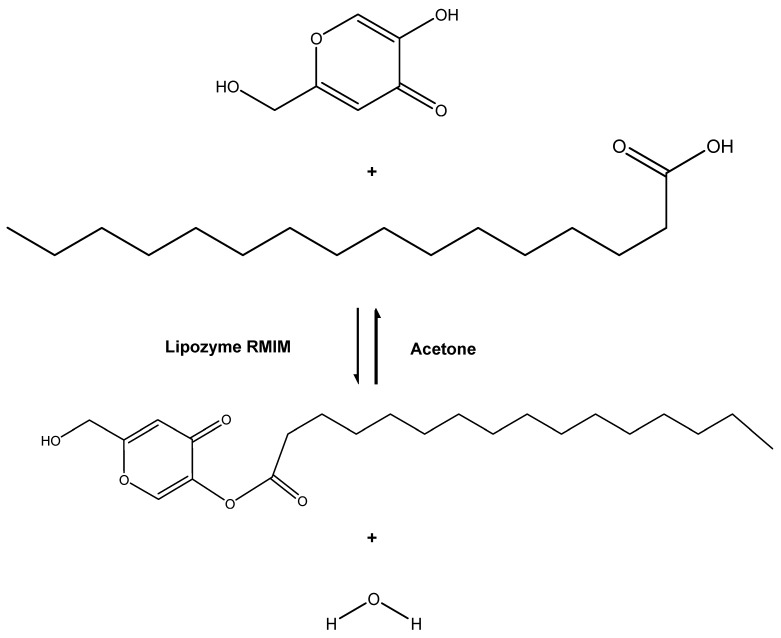
Reaction scheme of kojic acid derivative synthesis from palmitic acid and kojic acid in acetone using Lipozyme RMIM.

Based on our current knowledge, explanation of the rheological behavior of KAD in cosmetic formulation still has room for improvement. Until now, no statistical data is available on the stability of KAD in cosmetic formulation compared to kojic acid. Rheological studies can offer informative knowledge on how to produce cosmetic products including lotion for a new formulation. Therefore, in this study, a conversion of KAD using suitable solvent and saturated fatty acid as its acyl donor, was described (Figure 1), which also included the kinetics study for KAD synthesis. The corresponding rheological behavior and stability of a cosmetic cream containing KAD that was not mentioned in the previous study was also described.

In this study, Lipozyme RMIM was chosen as a model of biocatalysis. The use of properly immobilized and very improved enzymes enlarges the range of conditions. It has been proven that the suitable condition of immobilization is a non-trivial step in biocatalysis design and should be optimized to get maximum enzyme improvement [24]. Such a use has some kinetic advantages, such as saving the lag time usually required in these kinds of reactions [25]. However, this study was focused more on the physical treatment esterification process and compatibility of the solvent-substrate-product during the synthesis of KAD. Effects of other variables and support materials on the lipase-catalyzed esterification for KAD synthesis will be our priority as new potential projects in the future.

## 2. Results and Discussion

### 2.1. Kinetics Model

**Figure 2:**
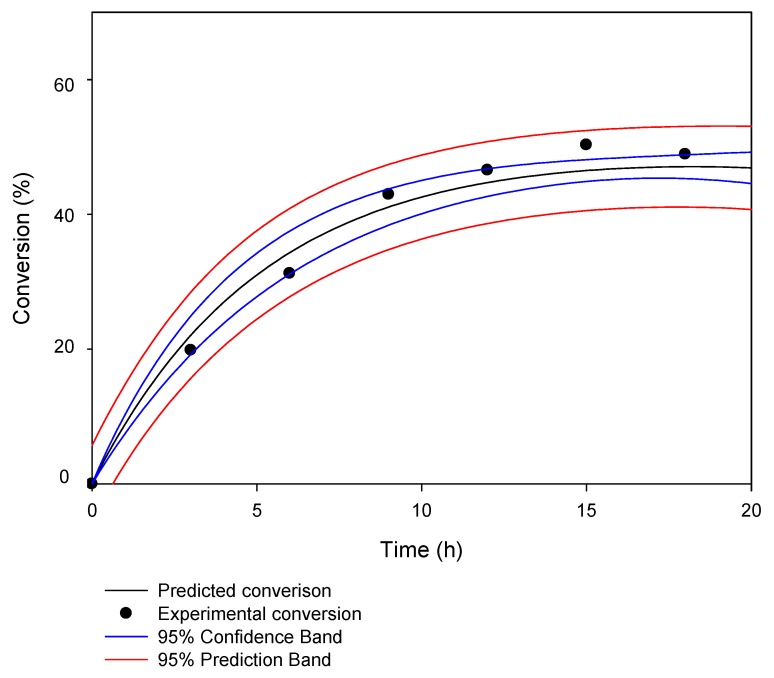
The conversion of kojic acid derivative (KAD) and the predicted model in acetone for 20-h reaction time where reaction temperature, substrate molar ratio, agitation and enzyme loading were fixed at 50 °C, 5:1 (fatty acid:kojic acid), 180 rpm and 1.25% (*w*/*v*), respectively.

The conversion of KAD synthesized by commercial Lipozyme RMIM increased with increasing reaction time (from 0 to 12 h) and the proposed model of fitness, Equation (4) (in the section of materials and methods). A good agreement between experimental and predicted conversion was observed (Figure 2) with kinetic parameters summarized in Table 1. The predicted yield fitted well with the experimental value and the statistical analysis indicated that the deviations were not significant at a probability of 0.05. The conversion (%) of KAD had increased within the first 12 h and a maximum value of 49.1% was observed before equilibrium. This further suggests that time course can pinpoint the adequate time period necessary to obtain suitable conversion and yields. During esterification, forward reaction was greater than reverse reaction until equilibrium. At equilibrium, the rate of the forward reaction was seen equal to the rate of the reverse reaction while the concentrations of substrates (palmitic acid and kojic acid) and products (KAD) had changed. The decrease in conversion may be due to lipase desorption during operation, which in return decreases the enzyme activity. Many substrates/products of lipases have some detergent character (free fatty acids) and may facilitate undesired lipase desorption during operation [26,27]. In order to improve the performance of KAD synthesis, polyethylenimine (PEI), specific encapsulant and cryoprotectant may be considered to modify enzymes as it might improve many enzyme properties such as stabilization versus organic solvents or oxygen, radical scavengers, heat and stabilization of multimeric structures [28,29].

### 2.2. Organic Solvent Compatibility

**Figure 3:**
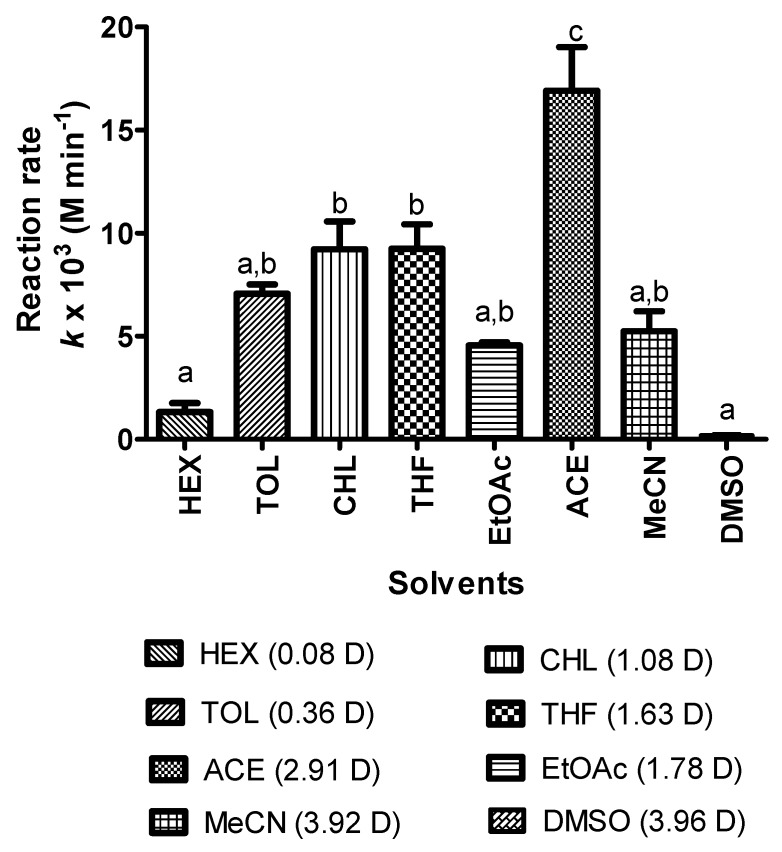
The rate of reaction of KAD synthesis in organic solvents with various dipole moments (D) where the reaction time, temperature, substrate molar ratio, agitation and enzyme loading were fixed at 12 h, 50 °C, 5:1, 180 rpm and 1.25% (*v*/*v*), respectively. Letters a, b, and c indicate significant difference at *p* <0.05 with Bonferroni’s multiple comparison test. Note: HEX: Hexane; TOL: Toluene; CHL: Chloroform; THF: Tetrahydrofuran; EtOAc: Ethyl acetate; ACE: Acetone; MeCN: Acetonitrile; DMSO: Dimethyl sulfoxide.

Regarding the polarity value, different types of solvents were evaluated for substrate and enzyme compatibility and it was found that acetone, (CH₃)₂CO, was a suitable solvent in the synthesis of KAD derivative. The true selection of the organic solvent is important due to the negative effect of lipase activity in organic media reported in the literature [28]. Figure 3 shows the pattern of the reaction rate (the average rate within 12 h) regarding the polarity of several solvents based on dipole moments (D) (acetone, 2.91 D; acetonitrile, 3.92 D; ethyl acetate, 1.78 D; tetrahydrofuran, 1.63 D; dimethyl sulfoxide (DMSO), 3.96 D; toluene, 0.36 D; chloroform, 1.04 D; hexane, 0.08 D). Previous studies have demonstrated that the partition-coefficient or Log *P* is important in explaining the correlation of the solvent with the concentrations ratio of hydrophilic kojic acid and hydrophobic fatty acids in these two immiscible phases. In general, the rate of reaction decreased at the highest log *P* (hexane, 4.66), but increased at the lowest log *P* (toluene, 3.34; chloroform, 1.97; ethyl acetate, 0.90; tetrahydrofuran 0.59; acetone −0.42; acetonitrile −0.19). The highest rate of KAD synthesis of 0.03 mmol·h^−1^ was obtained in acetone with Log *P* of −0.42 (polarity index, 6.2).

A high reaction rate was observed in acetone (polarity index, 6.2) compared to other organic solvents. Kojic acid is sparingly soluble in non-polar solvents, which may result in low yield of KAD synthesis. In this study, polar solvent (acetone) was selected due to being a suitable medium for enzyme activity and the compatibility with substrates and products. Solvents of suitable Log *P* showed the increased rate of KAD synthesis, which might be due to the suitable microenvironment to maintain a three-dimensional structure of the active site during enzymatic reactions. On the other hand, low reaction in ethyl acetate might be due to solvent-enzyme incompatibility which may disrupt 3D conformation and RMIM carrier. One more factor may also be contributed by ethyl acetate since this solvent was reported as a substrate for conversion during biocatalyst reactions [30].

In addition, some solvents, including DMSO and dimethylformamide (DMF), have inhibitory effects on enzyme activity and stability (Novozym^®^ 435) [16,17]. On the other hand, the effects of solvents on the p*K* value of an acid may also influence the synthesis of KAD. Kojic acid has an acid dissociation constant of p*K*_a_ of 7.7 at 25 °C. Density functional calculation showed that kojic acid is a good nucleophile that can donate a pair of electrons to form a new covalent bond (ester bond) [31]. The higher the p*K*_a_ of the conjugate acid, the more reactive it is as a nucleophile [31,32]. In this study, acetone had increased p*K*_a_ values of the substrates at a greater p*K*_a_ value compared to other organic solvents allowing a high reaction rate [32]. The solubility of the acids was found to increase with alcohol content and solvents, which is mainly due to electrostatic interactions that contribute to the p*K*_a_ value [32]. Another factor influencing the rate of conversion is the viscosity of the solvent as it may affect interaction, contact and migration of the substrate to the catalytic site of the enzyme. Solvents such as DMSO (1990 μPa·s), chloroform (786 μPa·s), toluene (560 μPa·s) and acetonitrile (461 μPa·s) are viscous compared to solvents like acetone (366 μPa·s). Therefore, in this study, the low viscosity of acetone meant that it was selected as the suitable solvent in KAD synthesis.

### 2.3. Biocatalyst Loading and Reaction Temperature

**Figure 4:**
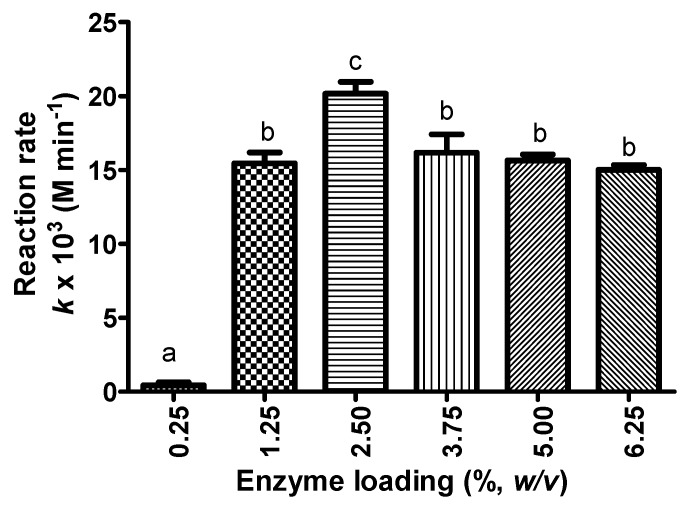
The rate of reaction of KAD synthesis in acetone using different enzyme loading (%, *w*/*v*) where reaction time, temperature, agitation, and substrate molar ratio were fixed at 12 h, 50 °C, 180 rpm and 5:1 (fatty acid:kojic acid), respectively. Letters a, b and c indicate significant difference at *p* < 0.05 with Bonferroni’s multiple comparison test.

The highest rate of reaction (20.17 × 10^−3^ M·min^−1^) was obtained using 2.5% (*w*/*v*) of Lipozyme RMIM (Figure 4). The Bonferroni’s Multiple Comparison Test illustrated that the reaction rate (average rate within 12 h) of KAD synthesis when using 2.5% enzyme loading was significantly different than 0.25% and 1.25%, respectively, which is in agreement with a typical biocatalyst reaction. However, further increase in the enzyme loading above 2.5% (*w*/*v*) resulted in a decrease of reaction rate. In this case, the enzyme molecules may be packed together with some intermolecular interactions that may be produced [33]. The rate of reaction was not significantly different at enzyme loading of 3.75–6.25%. Low enzyme loading may not be sufficient for KAD conversion within a specified period of time while higher loading may impose diffusion limitations on the system due to the formation of enzyme crowding. Moreover, it was discovered that when the support was fully loaded with some lipases, the stability of the enzyme decreased under certain conditions [34]. This phenomenon may affect the final performance of the immobilized enzyme, in a positive or a negative way [33]. The negative effect was explained by interactions between vicinal partially unfolded enzyme molecules (perhaps exhibiting hydrophobic pockets) that prevented the recovery of the enzyme activity when measuring the enzyme activity under mild conditions during inactivation experiments [33].

**Figure 5:**
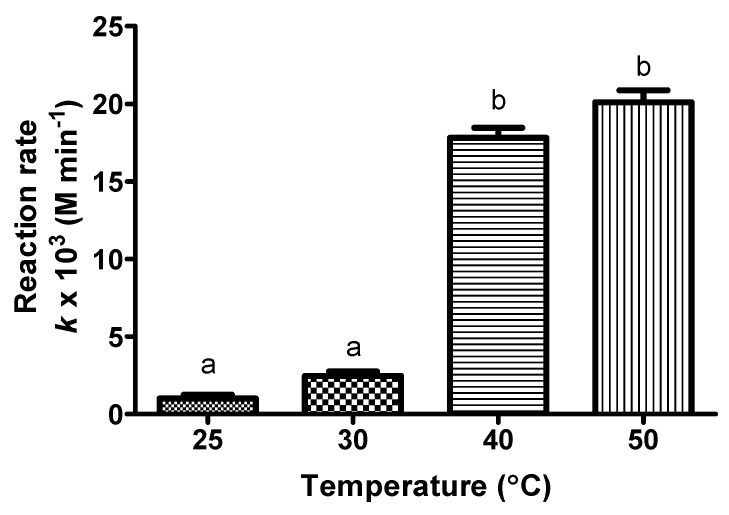
The rate of reaction of KAD synthesis in acetone at various reaction temperatures where reaction time, substrate molar ratio, agitation, and enzyme loading were fixed at 12 h, 5:1 (fatty acid:kojic acid), 180 rpm and 1.25% (*w*/*v*), respectively. Letters a and b indicate significant difference at *p* <0.05 with Bonferroni’s multiple comparison test.

On the other hand, temperature is also crucial for the conversion where the rate of reaction (average within 12 h) increases with increasing reaction temperature (Figure 5). The temperature over which the enzyme performed efficiently was 50 °C with a reaction rate of 19.56 × 10^−3^ M·min^−1^ owing to the lipases with their high stability, wide specificity coupled to a strict selectivity or specificity [35]. However, based on the Bonferroni’s multiple comparison test, the reaction rate at 40 °C and 50 °C was not significantly different. The esterification above 50 °C cannot be carried out due to the acetone boiling point of 56 °C and the enzyme desorption that may also occur under drastic conditions including high temperature [36], along with some substrates or products that may even facilitate enzyme release (free fatty acid, monoacetin or monobutyrin) [37]. The coating of the immobilized lipases with PEI, dextran sulphate and specific encapsulant revealed itself as a powerful tool to improve enzyme stability [26,29]. This is because it may occur via intermolecular physical crosslinking [26].

Poor performance was observed in low reaction temperatures (25 °C and 30 °C) due to the presence of high steric hindrances for the reaction with enzyme groups that involve only a minimum number of nucleophiles of the enzyme surface [38]. In addition, the tendency of palmitic acid to become solid during the reaction was observed, which may cause mass transfer limitation. As the reaction rate is the average rate within 12 h, the effect of equilibrium conversion at different temperatures was then one of the main reasons. Once the temperature of the equilibrium increased, the reaction rate of forward and reverse direction was also seen to increase. In this process, the substrates used more energy and increased the rate of endothermic reaction more than the exothermic reaction until equilibrium was reached [39]. The Bonferroni’s multiple comparison test showed a significant difference in reaction rate at low temperatures (25 °C and 30 °C) compared to high temperatures (40 °C and 50 °C). Reaction temperature is a crucial factor that enhances the catalytic interaction, reduces the viscosity and improves the solubility of substrates/products in the reaction medium [40].

**Figure 6:**
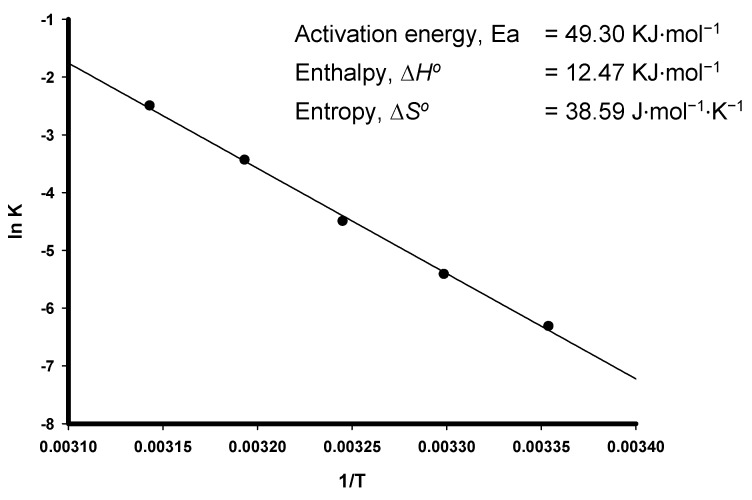
Activation energy and thermodynamic parameters of kojic acid derivative synthesis.

By applying the Arrhenius equation and plotting ln K versus 1/T as shown in Figure 6 for the forward reaction, the activation energy from the slope of the graph displayed a value of 49.30 kJ·mol^−1^, whereas the two thermodynamic parameters in the process estimated the enthalpy and entropy at 12.47 kJ·mol^−1^ and 38.59 J·mol^−1^K^−1^, respectively (calculated using the Van’t Hoff equation). In thermodynamic systems, at constant pressure, the reaction energy is equal to the change in the enthalpy (ΔH) of the chemical reaction. In endothermic reactions, the ΔH is positive, thus indicating that the heat is absorbed to break down the bond known as the activation energy (E_a_), a minimum amount of energy to allow substrates to undergo chemical transformation during a collision between substrates. In endothermic reactions, a larger E_a_ indicates that the reaction is more sensitive to the changes in temperature (ΔT). Thus, with increasing temperature, higher reaction rate can be observed in endothermic systems compared to exothermic systems. At low temperature, the reaction is slow due to the low occurrence of collision between substrates so that the chance of producing a new bond is low. However, at high temperatures, where the reaction is fast, the number of collisions increases with many new bonds being produced (KAD) [39].

The use of organic solvent system is believed to minimize hydrolysis and the thermodynamic equilibrium can be shifted towards synthesis [41,42,43]. However, these conditions must be compatible with substrate and product stability and solubility to shift the thermodynamic equilibrium in the desired direction, making their design very challenging [44]. Thermodynamic systems show a “switchlike” catalyst continuing in one direction until at least one reactant has passed completely into solution. On the other hand, in a more complex strategy, the kinetically controlled synthesis is another option in spite of its many practical disadvantages such as the need for activated acyl donors and the transient nature of synthetic yielding of a large excess of nucleophile (often, the more expensive and instable components [23]).

### 2.4. Fatty Acid Concentration

**Figure 7:**
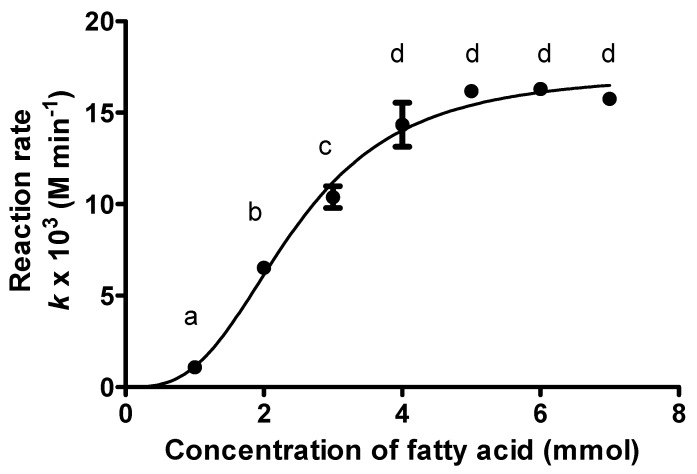
The yield of KAD synthesis in acetone with an increasing molar concentration of fatty acid (kojic acid is kept at 1 mol) where reaction time, temperature, agitation and enzyme loading were fixed at 12 h, 50 °C, 180 rpm and 1.25% (*w*/*v*), respectively. Letters a, b, c and d indicate significant differences at *p* < 0.05 with Bonferroni’s multiple comparison test.

The rate of reaction was gradually increased with increasing fatty acid concentration where kojic acid concentration was kept constant (Figure 7). Fatty acid is a suitable limiting substrate due to its ability to solidify at low temperature, low cost and to be easily removed during the purification process as compared to kojic acid [7]. The ratio of 5:1 palmitic acid to kojic acid was the optimal molar ratio that resulted in the highest reaction rate of 16.11 × 10^−3^ M·min^−1^, whereas higher fatty acid concentration (more than 5 mmol) showed a slight decreased. Increasing palmitic acid concentration beyond this point may not only provide excess palmitic acid, but also hinder the rate of reaction between enzyme and substrates. In addition, the high palmitic acid concentration has caused an increase in viscosity of the liquid phase that consequently reduced the degree of mixing, which may eventually introduce potential trouble to the downstream and purification processes. Even though higher concentrations of acyl donor are usually preferred for esterification in an organic solvent [45], it is also well known that high amounts of acid can disturb the microenvironment around the enzyme as the acids exhibit inhibitory action, thus decreasing the rate of the reaction [46,47,48]. Very high fatty acids in the reaction system could result in more fatty acids partitioning into the surrounding water shell and limit the access of substrates into the interface, hence reducing substrate-enzyme binding [48]. Previous studie have shown that the esterification rate of wax ester catalyzed by Lipozyme RMIM decreased at high hexanoic acid and octanoic acid concentrations [17,43]. Besides, it was reported that a competitive enzyme inhibition by butyric acid during esterification reactions by *Rhizomucor miehei* lipase led to the decrease in reaction rate [17,19]. Another study has reported that the esterification reaction by Lipozyme RMIM was also a substrate inhibited by lauric acid at some concentration [23].

### 2.5. Yield Optimization Using Response Surface Methodology

#### 2.5.1. Model Fitting, Analysis of Variance and Regression Analysis

Coded and actual levels of variables for response surface experimental design are represented in Table 2. The design matrix of the actual 30 experiments, together with the actual and predicted yield are illustrated in Table 3. From the analysis of variance (ANOVA) (Table 4), the model F-value is 16.09 with Prob > F-value < 0.0001, implying that the model was significant. The coefficient of determination (R^2^) of the model was 0.8786, indicating that the model is suitable to represent the relationships among parameters studied. Regression analysis of each set of data would result into the generation of the corresponding sets of coefficients for the development of a model equation (Table 5). Most of the non-significant terms were eliminated on the basis of Prob > F values larger than 0.05, not counting those required to support hierarchy (all linear terms), in order to come up with a final refined model. The reduced model for predicting the percentage yield of esterification of kojic acid (KA) and palmitic acid (PA) catalyzed by Lipozyme RMIM are thus shown in Equation (1).

Yield = 53.53 + 9.05X_1_ + 6.25X_2_ + 4.57X_3_ + 2.71X_4_ + 3.37X_2_X_3_ − 5.46X_2_X_4_ + 4.12X_3_X_4_ − 5.45X_2_^2^ − 4.13 X_3_^2^ (1)

The regression statistics showed that the adjusted R^2^ of 0.8240 was in reasonable agreement with its respective model R^2^. “Adequate precision” calculates the signal to noise ratio. A ratio larger than four is desirable. Thus, the ratio of 14.911 indicates an adequate signal for the model. Equation (1) was derived from regression analysis of the dataset as designed through the central composite rotatable design (CCRD) experimental method in Design Expert software (Stat-Ease, Inc., Minneapolis, MN, USA), and was then used to assist plotting of the response surfaces. Two parameters were plotted at any one time on the X_1_ and X_2_ axes, respectively, with the other two remaining parameters set at their center point values (coded level: 0). Fitting the data to the various mathematical models (linear, two factorial, quadratic and cubic) and their subsequent ANOVA analysis have shown that the KAD esterification reaction was suitably described by a quadratic model. There was only a 0.01% possibility that a ‘model F-value’ this large might occur due to noise. The non-significant lack-of-fit test (F-value = 1.48) which was comparative to the pure error of the experiments indicated that the quadratic model was suitable to represent the whole experimental data [49]. The model (Equation (1)) then can be used to navigate the design space.

#### 2.5.2. Response Surface Analysis

**Figure 8:**
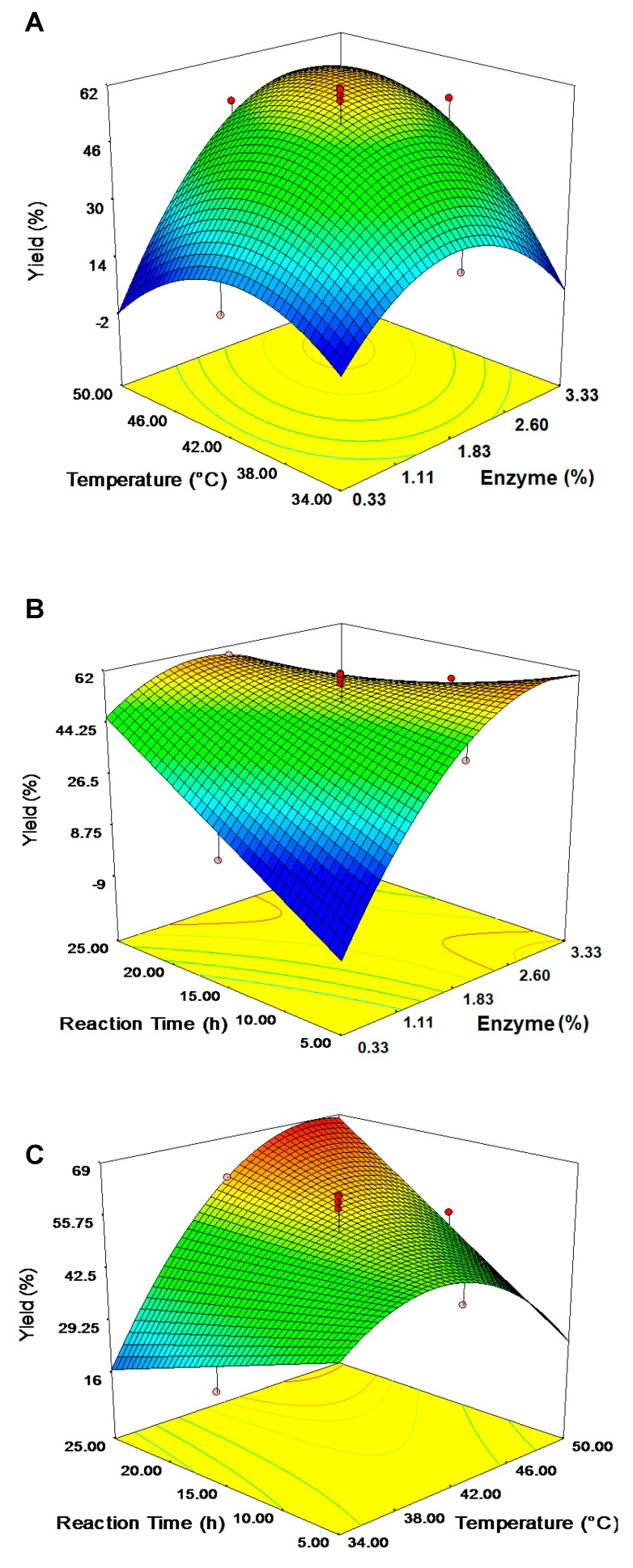
Surface plot; (**A**) Reaction temperature and amount of enzyme (X_2_X_3_) against yield (substrate ratio 5:1; reaction time 15 h); (**B**) Reaction time and amount of enzyme (X_2_X_4_) against yield (substrate ratio 5:1; temperature 42 °C); and (**C**) Reaction time and reaction temperature (X_3_X_4_) against yield (substrate ratio 5:1; amount of enzyme 1.83%, *w*/*v*).

Figure 8A shows the interactive effect of enzyme loading and reaction temperature (X_2_ X_3_) as a function of KAD yield. A response surface plot was generated with a PA to KA ratio fixed at 5:1 for a 15-h reaction period. The percentage yield of KAD (58.0%) was significantly improved with increasing reaction temperature and enzyme loading. However, no remarkable increase in the percentage yield was observed as the reaction time was extended for over 15 h. The interaction effect of enzyme loading and reaction time (X_2_ X_4_) on KAD yield is shown in Figure 8B. The esterification reactions of KA and PA to synthesize KAD were performed as the other two parameters were set at their center point values: temperature at 42 °C, and substrate ratio of 5:1. Figure 8C depicts the effects of reaction temperature and reaction time (X_3_ X_4_) on the esterification of KAD at the substrate ratio of 5:1 and enzyme loading of 1.83% (*w*/*v*). Yield of KAD was significantly increased with increasing reaction temperature. However, yield was only increased to a certain extent of temperature and then decreased regardless of the time of reaction. Many lipase-catalyzed esterification systems exhibit the type of surface plot such as in Figure 8, most popularly known as the dome shape [40]. 

Results from response surface analysis show that the increase in yield of KAD was observed with increasing enzyme loading at an earlier reaction time. However, as the reaction time was prolonged, the yield of KAD obtained was relatively constant at high enzyme loading. In the esterification reaction, enzyme loading greatly influenced a total reaction time required to achieve the desired conversion [40]. This observation is referred to as the inverse proportionality between reaction time and enzyme loading [50]. Generally, minimal use of enzyme is preferred since enzyme can be quite costly.

#### 2.5.3. Optimization of Esterification and Model Verification

The optimization function of Design Expert^®^ software was then used to obtain a solution with high desirability in order to predict the optimal condition for lipase-catalyzed synthesis of KAD (ratio PA:KA, 6.74; enzyme loading, 1.96% (*w*/*v*); reaction temperature, 45.9 °C; reaction time, 20 h). The esterification reaction was then conducted under the recommended parameters and the resulting response was compared to the predicted value. The response of reaction yield (64.47%) was comparable to the predicted value of 69.25%. Based on the optimum condition obtained, high product concentration as well as reduction of total operational cost may be achieved.

### 2.6. Stirred Tank Reactor

**Figure 9:**
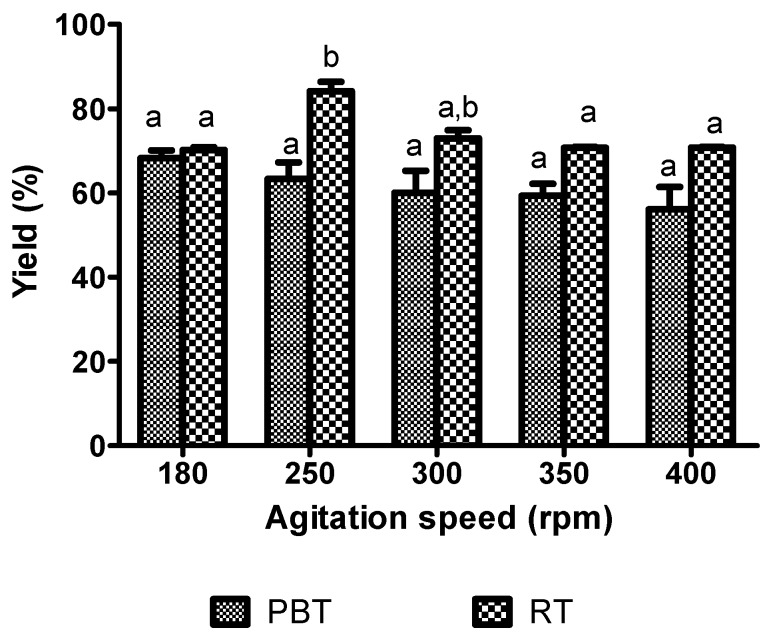
Effect of agitation speed on the percentage yield of kojic acid palmitate by using different impeller designs in a 0.5-L stirred-tank reactor. (Working volume: 0.2 L, substrate ratio: 6.74, enzyme loading: 1.96% (*w*/*v*), reaction temperature: 45.9 °C, reaction time: 20 h). Letters a and b indicate significant difference at *p* <0.05 with Bonferroni’s multiple comparison test. RT: Rushton turbine; PBT: pitch-blade turbine.

The yield of KAD synthesis in the 0.5-L stirred tank reactor (STR) (68.24 to 70.22%) when equipped with that of the two impeller design (RT and PBT) was comparable to the shake flask system (64.47%) when rotating at the same agitation speed (180 rpm) and subjected to Response Surface Methodology (RSM)-optimized condition. The performance of the KAD synthesis was further studied in terms of its yield by increasing the agitation speed of Rushton turbine (RT) and pitch-blade turbine (PBT) (Figure 9). By using an RT impeller, the yield of KAD was significantly increased at 250 rpm compared to 180 rpm but no significant difference was observed at a higher setting of 300–400 rpm as determined using Bonferroni’s multiple comparison test. On the other hand, lower yield was observed for the PBT impeller as compared to RT at any agitation speed. RT impeller showed a remarkable performance with the highest yield of 84.12% at 250 rpm.

Generally, the performance of KAD synthesis can be potentially improved through a suitable design of an impeller. In the STR system, the quality of flow generated during the reaction mainly depends upon the impeller design [51]. An increase in agitation speed can cause a substantial increase in a specific interfacial area between the substrate and the enzyme by reducing the droplet size [52,53]. For agitations above 250 rpm, the reaction performance was decreased due to the adverse shear effect. At low agitation speeds, poor mixing leads to mass or heat transfer problems as well as inhomogeneous enzyme distribution, both of which caused possible yield reduction [49]. On the other hand, at a very high agitation speeds, enzyme particles can be exposed to surface denaturation leading to a decrease in reaction performance [54]. A good reaction yield (95.8%) was previously observed in enzymatic hydrolysis of pre-treated spruce and giant reed by using a radial flow RT impeller as compared to axial flow elephant ear impellers [53].

The use of a 0.5-L STR to study the effect of impeller design and agitation speed on the mixing performance and the lipase-catalyzed esterification process for the synthesis of kojic acid ester may provide some basic knowledge and it can be used for further studies on bigger scale STR systems. Therefore, in future undertakings, in order to generate more useful data and information for scaling-up purposes, the process shall be carried out in large scale STR (>10 L) equipped with multiple impeller. In studies such as those previously reported, more detailed effects of mixing conditions measured as mixing time, fluid flow pattern, shear effect and hydrodynamic conditions could be determined [55]. The stability of the immobilized enzyme particles during the reaction subject to different degree of mixing and shear effect shall also be investigated.

Prior research on the synthesis of KAD has been successfully carried out at the laboratory scale using a shake flask and screw-capped test tubes where mixing and mass transfer limit the reaction [13]. Although various types of reactors have been used for the production of various products in large scale, reports on the use of a stirred tank reactor for KAD production are scarce. Results of this study have demonstrated the yield (84.12%) obtained in enzymatic synthesis using STR was substantially higher than those previously reported in the literature for KAD (40%) [7]. In this study, the enzymatic esterification for the synthesis of KAD was carried out as a batch process. The yield and productivity of the process may be improved by using other modes of bioreactor operation such as continuous processes [9]. The use of other types of bioreactors, such as packed bed, may also be useful for continuous process operation to improve the esterification performance [56]. Bioreactors with non-mechanical agitation systems may provide better enzyme protection than that usually observed in a stirred tank reactor by mechanical damage due to the high shear created by the use of the impeller [57,58]. The efficient and low-cost method for the separation and purification of KAD from the remaining substrates and other by products should be developed. Separation or extraction of KAD from the reaction mixture may be performed by liquid-liquid extraction methods while crystallization processes may be used to remove the remaining reactants including alcohol and lipid impurities in the esters [40]. Various solvents such as ethanol may be used to remove the remaining unreacted alcohol in the mixture.

### 2.7. Thermogravimetric and Differential Scanning Calorimetry Analysis

The melting point and thermal degradation of palmitic acid, kojic acid derivative and kojic acid is shown in Table 6. Melting point of KAD was significantly higher than palmitic acid due to the presence of both kojic acid and palmitic acid in KAD molecules. Based on DSC analysis, thermal degradation of KAD was also very high at 225.50 °C showing its stability at high temperature.

### 2.8. Rheology

**Figure 10:**
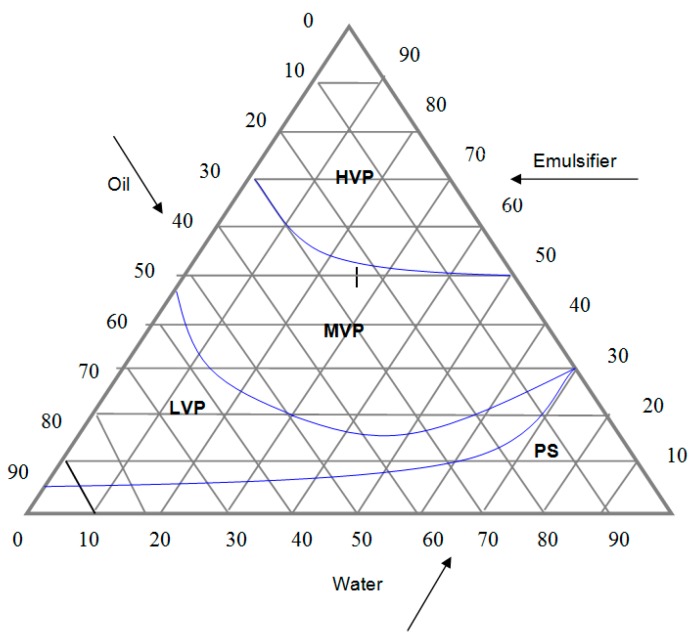
A standard phase diagram of formulation using difference percentage (*w*/*w*) of palm oil, emulsifier and distilled water. HVP: High viscous phase; MVP: Medium viscous phase; LVP: Low viscous phase; PS: Phase separation.

Figure 10 shows the phase diagram for the different ratio of palm oil, emulsifier and water, which was used as the standard lotion (LS) formulation for the KAD (or kojic acid)-containing lotion. Region where low viscous phase (LVP) occurs, particularly at 20% oil, 60% water, 20% emulsifier (*w*/*w*) was selected for rheological comparison for LS, LS + kojic acid, and LS + KAD. Rheological measurements provide information on the physical behavior and stability of lotion, an emulsion formed from two immiscible liquid (oil and water). Physical stability is important to avoid lotion instability including flocculation, phase inversion, creaming, sedimentation, coalescence and Ostwald ripening. Few studies demonstrated a good relation of rheological and sensory properties including thickness, ease of spreading on skin and smooth flow from the bottle [59,60]. Table 7 shows the flow behavior (*n*) and consistency index (*K*) of several compounds includes pectin, xanthan gum, carboxymethyl cellulose (CMC), LS, LS + kojic acid, LS + KAD, and three commercial lotions (CLs) available on the market. All samples exhibit pseudoplastic behavior where *n* is less than 1. K, is a direct quantification of viscosity at a given rate of shear. The greater the value of K, the bigger the viscosity at a given shear rate. The formulated and commercial lotions were significantly not viscous (*K*_25 °C_ = 0.0224 to 3.2297) as compared to viscous control compounds such as pectin, xanthan gum, and CMC (*K*_25 °C_ = 12.8131 to 200.0598). All lotions were significantly different from palm oil where the *K* value of lotions was significantly larger than the *K* value of palm oil. In this study, LS + KAD showed less viscosity as compared to LS + KA at 25 °C. This is due to proper incorporation of lipophilic KAD into LS formulation than hydrophilic kojic acid. Less viscosity means that it can be applied on skin smoothly. LS + KAD, CL-Safi, and CL-Nivea showed similar *K*_25 °C_ value of 3.0277, 2.2866, and 0.6456, respectively, while producing *K*_40 °C_ value of 1.0206, 1.8331 and 0.8564, respectively.

**Figure 11:**
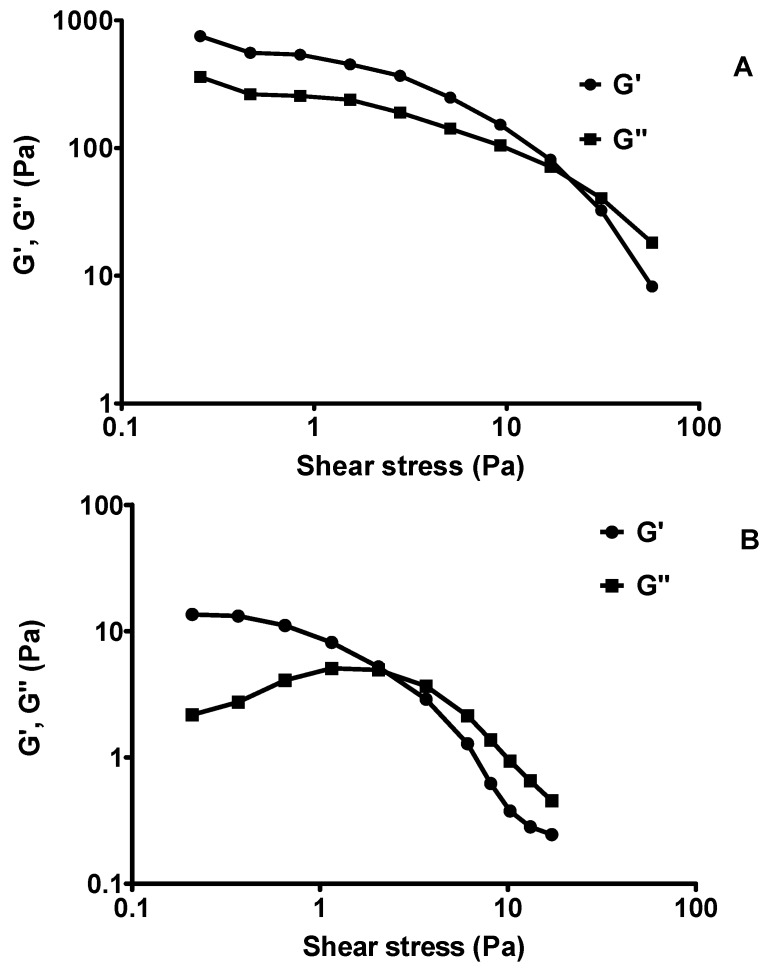

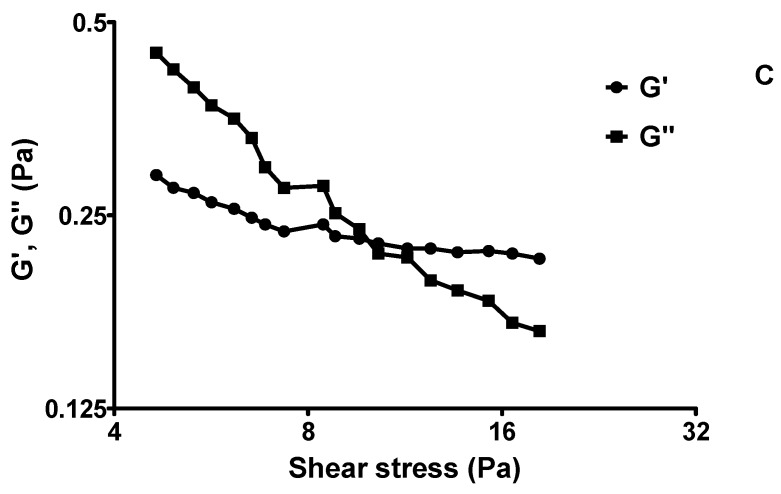
The storage modulus (G’) and the loss modulus (G”) during an amplitude sweep (oscillating measurement) for; (**A**) LS + 5% KAD; (**B**) CL_1_ Safi; and (**C**) CL_2_ Nivea.

These three lotions where further tested for amplitude sweep test (oscillation). In an amplitude sweep, the amplitude of the shear stress was varied while the frequency was kept constant where the storage modulus (G’) and the loss modulus (G’’) are plotted against the amplitude of the shear stress Figure 11. At low amplitude of the shear stress G’ and G’’ are steady, the sample structure is undisturbed and is said to be in a state of linear-viscoelastic. As the moduli start to decrease at a significant level, the structure is disturbed where the plateau value of G’ in the linear-viscoelastic state describes the inflexibility of the sample and if the G’ is larger than the G’’ as shown in Figure 11A,B, the samples LS + KAD and the commercial CL-Safi^®^ behave more like a viscoelastic solid. After the state of linear-viscoelastic, samples LS + KAD and CL-Safi^®^ behave more like a viscoelastic liquid (G” is larger than G’). The cross-over of G’/G” is taken as the yield point, as this indicates the transition from solid to liquid characteristics. The larger the difference between the moduli (G’ and G”), the difference of a pure fluid and solid state of the samples are more apparent. Unlike LS + KAD and CL-Safi^®^, the CL-Nivea^®^ (Figure 11C) showed viscoelastic fluid at the beginning and after the cross-over of G’/G”, it turns into viscoelastic solid. The amplitude sweep is essential to examine the linear viscoelastic region of a lotion that is a range of shear stress in which the formulation neither suffers from intense alterations on it molecular structure, nor is disrupted. Once a shear stress of the linear viscoelastic region is imposed in an oscillatory test, only the inter-molecular and inter-particle forces are evaluated. Using a sweep test, the G’ value is higher than G’’ it is indicative that the formulation is more elastic than viscous and it is described as more stable than formulations with G’’ values higher than G’ due to the fact that rate of recovery of its original structure is more rapid and efficient than the other formulations. They are also less vulnerable to the mass forces, which slows down or avoids phase separation and coalescence process of emulsions. So, the G’ values that are higher than G’’ in emulsions are a desirable feature, being an indicative of stability of the cosmetic formulation system [61].

## 3. Materials and Method

### 3.1. Materials

Immobilized lipase from *Rhizomucor miehei* (Lipozyme RMIM) was purchased from Novo-Nordisk, Denmark. Kojic acid crystal was obtained via aerobic fermentation using locally isolated strain as described previously [13]. Palmitic acid (99%) was purchased from Sigma-Aldrich (St. Louis, MO, USA). All other chemicals, solvents and reagents used in this study were of analytical grade and sourced from Merck, Germany unless otherwise stated. Lipozyme RMIM was immobilized using macro-porous Duolite ES 562 (a weak anion-exchange resin) based on phenol-formaldehyde copolymers, respectively [14,17,62]. The catalytic activity of RMIM was 5–6 Batch Acidolysis Units Novo (BAUN) is based on a reaction between high oleic sunflower oil and decanoic acid at 70–80 °C for 60 min.

### 3.2. Methods

#### 3.2.1. Synthesis of KAD

The reaction system in a 0.25-L shake flask consisted of 6 mmol palmitic acid, 1 mmol kojic acid, 30.0 mL acetone, and 0.55 g Lipozyme RMIM (Figure 1). The mixture was incubated at 50 °C using a horizontal water bath shaker. The agitation speed was set at 180 rpm and the reaction mixture was continuously reacted for 24 h. The reaction was then filter terminated with Whatman filter paper No. 1, removing Lipozyme RMIM from the reaction system, and the solvent was removed by rotary evaporator (BUCHI model R-220, Essen, Germany).

#### 3.2.2. Reaction Rate of the Synthesis of KAD

One-factor-at-a-time (OFAT) experiments were used to evaluate the reaction rate of the synthesis of KAD by varying one parameter at a time while keeping others fixed. The rate was studied in 0.25-L shake flasks placed in a shaking water bath and agitated at 180 rpm. The parameters selected for the esterification experiments were (i) reaction time (0, 3, 6, 9, 12, 15, 18, 21 and 24 h); (ii) reaction temperature (25, 30, 40 and 50 °C); (iii) enzyme loading (0.25, 1.25, 2.5, 3.75, 5 and 6.25% *w*/*v*); (iv) molar ratio of PA:KA (1:1, 2:1, 3:1, 4:1, 5:1, 6:1 and 7:1) and (v) partition-coefficient (Log *P*) of polar and non-polar solvent (i.e., ethyl acetate, 0.90), polar aprotic (i.e., acetonitrile −0.19, acetone −0.42, tetrahydrofuran 0.59, toluene 3.34, chloroform 1.97, DMSO −1.4), non-polar (i.e., hexane 4.66). Samples were taken at the end of reaction for further analysis.

#### 3.2.3. Kinetic Equation

A kinetic equation that obeys a two-substrate reversible reaction mechanism was considered (Equation (2)) and the mechanism is as follows;

(2) $$S_{k}+S_{p}\overset{k_{1}}{\underset{k_{-1}}{⇌}}P+W$$

where *S_k_* is kojic acid, *S_p_* is palmitic acid, *P* is KAD, *W* is water, *k*_1_ is forward reaction rate, *k*_−1_ is reverse reaction rate.

Then, the reaction rate I equation was expressed as in Equation (3):

(3) $$r=-\frac{\Delta S_{p0}}{\Delta t}=k_{1}[S_{p0}][S_{k0}]-k_{-1}[P][W]$$

Upon optimization, the integrated rate equation associated to this mechanism would result in the form of a parametric equation (Equation (4)), which contains two parameters (*α* and *β*) and the constant rate of the direct reaction (*k_1_*) can be calculated using Equation (5) [63]:

(4) $$X=\frac{\alpha (1-e^{\beta \cdot t})}{e^{\beta \cdot t}(1-\{1+([S_{p0}]/[S_{k0}])\}\alpha )+1}$$

(5) $$k_{1}=\frac{\beta }{\left[\left[S_{p0}]+[S_{k0}]-(2\cdot [S_{k0}]/\alpha \right)\right]}$$

where [*S_p_*_0_] and [*S_k_*_0_] are the concentrations of the palmitic acid and kojic acid, respectively, *X* is conversion (%), *α* is parameter related to equilibrium conversion (*K*), *β* is parameter related to rate constant (*k*), t is the reaction time.

The kinetic equation was fitted and performed by non-linear curve-fitting with a Marquadt–Levenberg algorithm model using Sigmaplot (version 11) software for the optimization. It was used as a search method to reduce the sum of squared deviations between the predicted and experimental values. The values predicted were then used to simulate the KAD conversion.

#### 3.2.4. Calculation of the Activation Energy (E_a_) and Thermodynamic Parameters

The *E_a_* of the reaction was calculated from the Arrhenius equation, where reaction rate is proportional to the *E_a_* (Equation (6)). Arrhenius equation is a formula for the temperature dependence of the rate constant, and thus rate of reaction. Additionally, the thermodynamic parameters of the reaction were estimated using the Van’t Hoff equation (Equation (7)). The Van’t Hoff equation describes the temperature dependence of the equilibrium constant assuming that the enthalpy of the reaction is constant as the function of temperature.

(6) $$\ln k=\ln k_{o}-\left(\frac{E_{a}}{R}\right)\left(\frac{1}{T}\right)$$

(7a) $$\frac{d\ln K}{dT}=\frac{\Delta H^{o}}{RT^{2}}$$

(7b) $$\Delta S^{o}=-\frac{\Delta H^{o}}{T}$$

where, *k* = reaction rate constant (M^−1^·min^−1^), *K* = equilibrium constant (M), *R* = gas constant (8.314 J·mol^−1^·K^−1^), *E*_a_ = activation energy (J·mol^−1^), *T =* absolute temperature (K), ∆*H^o^* = enthalpy (KJ·mol^−1^) and ∆*S^o^* = entropy (J·mol^−1^·K^−1^). ΔH is the standard enthalpy change of a reaction, the amount of heat absorbed or released in a reaction and it signifies whether the system is endothermic or exothermic. ΔS is the change in entropy of the reaction system driven by heat flow of the surrounding.

#### 3.2.5. Optimization Study Using Response Surface Methodology

Interactive effects of the important parameters to the esterification reaction of KA and PA were carried out in 250-mL shake flasks. A five-level-four-factor central composite rotatable design (CCRD) was computed using Design Expert^®^ Version 7.0.0. The design matrix of the actual 30 experiments was made up from a fractional design (Runs 1–16) combined with 6 center points for error estimation (Runs 25–30) and 8 axial points (Runs 17–24) where one variable is set at an extreme level (±2) while keeping others at their center point values. The reactions were catalyzed in acetone by various amounts of enzyme (from 0.33 to 3.33%, *w*/*v*) at different temperatures (34 to 50 °C) and reaction times (5 to 25 h). The ranges of PA to KA ratio were set from 1:1 to 9:1, where optimum final product concentration was observed in this range. Experiments were then conducted under the recommended parameters and the resulting responses were compared to those predicted by the software. For each model selected, a set of optimal reaction condition was generated using the numerical optimization function in the Design Expert^®^ software based on a desirability ranging from 0 (least desirable) to 1 (most desirable).

#### 3.2.6. Stirred Tank Reactor 

**Figure 12:**
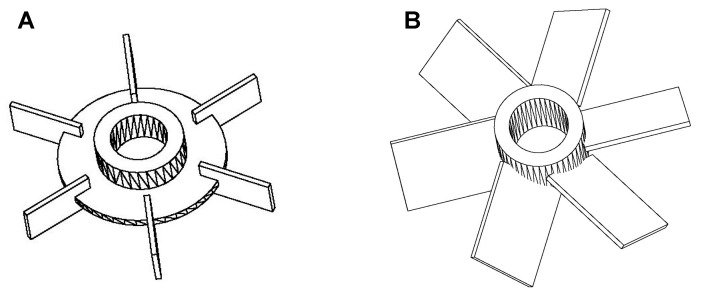
Impellers used for studying the agitation effect on KAD synthesis in 0.5 L STR; (**A**) Rushton turbine and (**B**) pitch-blades disc turbine (PBT).

The 0.5-L STR with a working volume of 0.2 L was used to synthesize the KAD. The STR consisted of a jacketed borosilicate glass vessel with a stainless-steel top-plate (without baffle). The top-plate consisted of openings for the introduction of enzyme and sampling purposes. Agitation was provided by an impeller immersed in the reaction medium at 2-cm height from the bottom of the vessel. The reaction temperature was controlled by circulating water from a water bath set at the required temperature into the jacketed vessel. A six bladed Rushton turbine (Figure 12A) and a six 45°-angled pitch bladed disc turbine impellers were used to study the effect of impeller designs on the basis of varying the agitation speeds from 180 to 400 rpm. The optimum substrate ratio of PA to KA (6.74:1) and enzyme loading (1.97% *w*/*v*) obtained from RSM prediction were used in this experiment.

#### 3.2.7. Calculation of Yield

Gas chromatography (Agilent 7890A) analysis of KAD samples were carried out using a non-polar Zebron-5HT capillary column (15 m × 0.53 mm × 0.15 µm) with nitrogen as a carrier gas. Temperature was started at 100 °C, (held for 1 min), and was programmed to increase at 15 °C/min up to 130 °C, (held for 1 min). Temperature was further increased at an even rate up to 150 °C (held for another 1 min). Oven temperature was then increased to 225 °C at 15 °C/min, (held for 1 min) and programmed to the final, 260 °C at 30 °C·min^−1^, (held for 1 min). Injector and detector temperatures were set at 340 °C and 350 °C, respectively. Product compositions were then quantified with 1, 2, 3-tributyrylglycerol as an internal standard. The amount of KAD obtained by GC analysis was calculated using Equation (8):

(8) $$C_{x}=\left(\frac{A_{x}}{A_{IS}}\right)\times \left(\frac{D_{Rf_{IS}}}{D_{Rf_{\infty }}}\right)\times C_{IS}$$

where *C_x_* = amount of component *X*; *C_IS_* = amount of internal standard; *A_x_* = area of component *X*; *A_IS_* = area of internal standard; *D_Rfx_* = detector response factor of component *X*; *D_RfIS_* = detector response factor of internal standard. The percentage yield of KAD is then defined using Equation (9):

(9) Yield=([KAD]KAwKAmw)×100

where [*KAD*] = concentration of KAD (mmol); *KA_w_* = weight of *KA* (g); *KA_mw_* = molecular weight of *KA* (g/mol) and yield expressed in percent (%).

#### 3.2.8. Purification and Characterization of KAD Compound

The crude extract was subjected to purification using a 21 × 1.5 cm column chromatography (17 cm height) containing a silica gel column using a mixture of hexane:ethyl acetate (71:29 *v*/*v*) as an eluent. The effluent was collected in a 20-mL sampling bottle. To confirm the purity, each fraction was spotted on a thin layer chromatography (TLC) aluminium sheet coated with silica gel G60F254 with hexane:ethyl acetate (71:29 *v*/*v*) as a developing solvent. The suspected pure ester was detected as a brown spot after being treated with iodine vapor. GC-MS analysis of KAD sample was performed using a Shimadzu Instrument, QP5050A spectrometry. Helium was used as a carrier gas at a flow rate of 1.5 mL·min^−1^. Column temperature was held at 50 °C for 3 min, elevated to 275 °C at 40 °C·min^−1^ and finally held for 25 min at 275 °C. Injector and detector temperatures were set at 340 and 350 °C, respectively. Proton NMR and carbon NMR (^1^H-NMR and ^13^C-NMR) spectra were obtained using JNM-ECA400 spectrometer in deuterated acetone. Tetramethylsilane was used as an internal standard. Spectrum analysis of KAD sample was recorded using FTIR Spectroscopy and potassium bromide (KBr) pellets for solid and paste samples while sodium chloride (NaCI) cell were used for liquid samples.

#### 3.2.9. Thermogravimetric and Differential Scanning Calorimetry Analysis

The study of decomposition for KAD was carried out using a thermo analyzer (TGA Q500 V20.13 Build 39) under N_2_ condition with a heating rate of 10 °C·min^−1^ from room temperature to 800 °C. On the other hand, the melting point study of KAD was evaluated using a differential scanning calorimeter (DSC Q20 V24.10 Build 122) under N_2_ atmosphere at a heating rate 10 °C·min^−1^ from 25 °C to 160 °C.

#### 3.2.10. Phase Diagram and Lotion Formulation 

A standard phase diagram was constructed based on different ratios of palm oil, emulsifier (beeswax) and water. The mixture of palm oil and emulsifier was continuously stirred for 60 min by homogenizer with an addition of water and heated at a controlled temperature of 40 °C before standing at 25 °C for 24 h to equilibrate the system. The phase systems formed were catalyzed for phase separation where the different regions were delineated in the phase diagram. The percentages of the three components were calculated after each new addition of distilled water to define the boundaries between the regions of the ternary phase diagram. By using the standard phase diagram, lotion containing either kojic acid or KAD was formulated on 100-g oil-based lotion basis. In this study, lotion was made of 20% oil, 60% water and 20% emulsifier where kojic acid (5% *w*/*w*) was dissolved in water and KAD (5% *w*/*w*) was dissolved in oil at 40 °C using a magnetic stirrer (500 rpm) on a IKAMAG hotplate stirrer for 30 min and homogenizer for 60 min. Then, the lotion mixture was stand down 25 °C for 4 h. The rheological stability was studied where both lotions were subjected to incubation temperature at 25 °C and 40 °C for 30 min.

#### 3.2.11. Rheology 

The melt rheological study of the samples were evaluated using a HAAKE™ MARS III Thermo Scientific Rheometer where the effect of shear rate, viscosity, shear stress, temperature were also studied. The measurements were carried out in the rotational mode and 20 mm parallel -plate with gap size of 0.5 mm. Every rheological study was performed at its respective temperature and the temperature was controlled by a water bath connected to the peltier system in the bottom plate. The sample was placed between the plates and the edges were carefully trimmed for precise measurement with an aluminium rod. The samples were allowed to equilibrate at respective temperatures for 3 min before each measurement. Shear stress-shear rate and viscosity-shear rate data of the samples were obtained at 25 °C and 40 °C. The flow experiments were conducted under steady-shear condition. The instrument was programmed for a set of temperature and equilibration followed by one-cycle shear in which the shear rate was increased linearly from 50 to 1000 s^−1^ in 2 min. The samples were measured in triplicate. All measurements were conducted in a normal atmospheric environment and consistency index (*K*) and flow behavior (*n*) was calculated using Ostwald–de Waele power-law equation which the shear stress, τ, given by:

(10) $$\tau =K{\left(\dot{\gamma }\right)}^{n}$$

where *K* is the flow consistency index (Pa·s*^n^*), $\dot{\gamma }$ is the shear rate or the velocity gradient vertical to the plane of shear (s^−1^), and *n* is the flow behavior index (dimensionless) represents an apparent or effective viscosity as a function of the shear rate (SI unit Pa·s).

Then, the samples were tested for amplitude sweep where the amplitude of the shear stress was varied, and the frequency was kept constant. The storage modulus (G′) and the loss modulus (G″) were plotted against the amplitude of the shear stress.

## 4. Conclusions

The results from this study suggest that the esterification of KA with PA mediated by Lipozyme RMIM can be properly carried out for the synthesis of KAD in a proper selected media. In a shake flask system, the relationship between the variables and responses in the synthesis of KAD has been adequately described by RSM and the 64.47% of esterification has been attained under this optimal condition. In the STR system, the degree of mixing had further influenced esterification with the highest yield of 82.14% obtained using RT agitated at 250 rpm. This enzymatic process has not only improved the lipophilic characteristics by the production of KAD, but also gave rise to the production of value-added derivatives from palm oil using potentially better operation system. The growing interest with the use of immobilized lipase guides the development of a new immobilization protocol. With few factors (where the enzyme will be used, potency of agitation and substrates used) taken into consideration, the use of the new immobilized lipase may improve the result. A proposed pseudoplastic behavior of the KAD mixture can be potentially used in lotion formulation.

## Figures and Tables

**Table 1:** Estimated kinetic rate constant of kojic acid derivative synthesis.

Kinetic Parameter	Value	Standard Error of Estimate
Coefficient *α*, parameter related to equilibrium constant, *K*	0.50	±0.00
Coefficient *β*, parameter related to rate constant, *k*	0.19	±0.01
Kinetic rate constant, *k*_1_ (M^−1^·min^−1^)	0.52	±0.03
*R*^2^	0.98	-

**Table 2:** Coded and actual levels of variables for response surface methodology (RSM) experimental design.

Variable	Symbol	Coded Level				
		−2	−1	0	1	2
		Corresponding Operation Value				
PA:KA (mmol)	X_1_	1	3	5	7	9
Enzyme loading (%, *w*/*v*)	X_2_	0.33	1.11	1.83	2.60	3.33
Temperature (°C)	X_3_	34.0	38.0	42.0	46.0	50.0
Reaction time (h)	X_4_	5	10	15	20	25

**Table 3:** Design matrix depicting actual and coded level combination and the corresponding response for a five-level, four-variable central composite rotatable designs.

Run	Actual Levels				Coded Levels				Response, Yield (%)	
	X_1_	X_2_	X_3_	X_4_	X_1_	X_2_	X_3_	X_4_	Actual	Predicted
1	3	1.10	38.0	10	−1	−1	−1	−1	27.4	23.4
2	7	1.10	38.0	10	1	−1	−1	−1	45.0	41.5
3	3	2.60	38.0	10	−1	1	−1	−1	42.3	40.1
4	7	2.60	38.0	10	1	1	−1	−1	58.9	58.2
5	3	1.10	46.0	10	−1	−1	1	−1	21.5	17.6
6	7	1.10	46.0	10	1	−1	1	−1	37.5	35.7
7	3	2.60	46.0	10	−1	1	1	−1	46.8	47.7
8	7	2.60	46.0	10	1	1	1	−1	61.8	65.8
9	3	1.10	38.0	20	−1	−1	−1	1	37.9	31.5
10	7	1.10	38.0	20	1	−1	−1	1	51.3	49.6
11	3	2.60	38.0	20	−1	1	−1	1	30.1	26.4
12	7	2.60	38.0	20	1	1	−1	1	38.0	44.5
13	3	1.10	46.0	20	−1	−1	1	1	43.0	42.1
14	7	1.10	46.0	20	1	−1	1	1	59.5	60.2
15	3	2.60	46.0	20	−1	1	1	1	48.7	50.5
16	7	2.60	46.0	20	1	1	1	1	66.0	68.6
17	1	1.83	42.0	15	−2	0	0	0	20.9	35.4
18	9	1.83	42.0	15	2	0	0	0	69.4	71.6
19	5	0.33	42.0	15	0	−2	0	0	10.1	19.2
20	5	3.33	42.0	15	0	2	0	0	50.3	44.2
21	5	1.83	34.0	15	0	0	−2	0	21.5	27.9
22	5	1.83	50.0	15	0	0	2	0	49.4	46.2
23	5	1.83	42.0	5	0	0	0	−2	42.6	48.1
24	5	1.83	42.0	25	0	0	0	2	58.6	59.0
25	5	1.83	42.0	15	0	0	0	0	61.1	53.5
26	5	1.83	42.0	15	0	0	0	0	57.7	53.5
27	5	1.83	42.0	15	0	0	0	0	59.3	53.5
28	5	1.83	42.0	15	0	0	0	0	50.3	53.5
29	5	1.83	42.0	15	0	0	0	0	60.6	53.5
30	5	1.83	42.0	15	0	0	0	0	48.8	53.5

**Table 4:** Analysis of variance (ANOVA) and R-squared (R^2^) analysis of biocatalysis of lipophilic kojic acid derivative (quadratic model).

	Source	Sum of SQUARES	Degree of Freedom	Mean Square	F-Value	Prob > F
ANOVA	Model	5715.73	9	635.08	16.09	<0.0001 ^a^
	Residual	789.47	20	39.47	-	-
	Lack-of-fit	644.23	15	42.95	1.48	0.3520 ^b^
	Total	6505.20	29	-	-	-
Regression Statistics	R^2^	0.8786	-	-	-	-
	Adjusted R^2^	0.8240	-	-	-	-
	Adequate Precision	14.9110	-	-	-	-
	Standard deviation	6.2800	-	-	-	-

^a^ Model F-value is significant at “Prob > F” less than 0.05; ^b^ Lack-of-fit value is not significant relative to the pure error.

**Table 5:** Values and significance of regression coefficients for lipophilic kojic acid derivative reaction (quadratic model).

Coefficients of Models	Yield (%)	
	Value	Prob > F
Intercept	53.53	-
X_1_	9.05	<0.0001 ^a^
X_2_	6.25	<0.0001 ^a^
X_3_	4.57	0.0020 ^a^
X_4_	2.71	0.0473 ^a^
X_2_ X_3_	3.37	0.0441 ^a^
X_2_ X_4_	−5.46	0.0024 ^a^
X_3_ X_4_	4.12	0.0163 ^a^
X_2_^2^	−5.45	0.0002 ^a^
X_3_^2^	−4.13	0.0022 ^a^

^a^ Significant at “Prob > F” of less than 0.05; Where; X_1_ = Substrate ratio, X_2_ = Enzyme amount, X_3_ = Temperature, X_4_ = Reaction time.

**Table 6:** The melting point and thermal degradation of palmitic acid, kojic acid derivative (KAD) and kojic acid.

Compounds	Melting Point	Thermal Degradation
Palmitic acid	63.44 °C	172.47 °C
KAD	135.11 °C	225.50 °C
Kojic acid	152.78 °C	243.32 °C

**Table 7:** Flow behavior (*n*) and consistency index (*K*) of viscous compounds (pectin, xanthan gum, carboxymethyl cellulose), palm oil, and commercial lotion (CL). Note: KA: Kojic acid; KAD: Kojic acid derivative.

Compounds	Flow Behaviour (*n*)		Consistency Index (*K*)	
	25 °C	40 °C	25 °C	40 °C
10% (*w*/*v*) pectin	0.48 ± 0.01	0.60 ± 0.01	12.81 ± 3.23	5.29 ± 0.38
10% (*w*/*v*) xanthan gum	0.16 ± 0.06	0.18 ± 0.01	49.73 ± 12.04	37.85 ± 1.78
10% carboxymethyl cellulose	0.10 ± 0.03	0.22 ± 0.01	200.06 ± 34.81	138.96 ± 9.62
Palm oil	0.86 ± 0.02	0.86 ± 0.02	0.03 ± 0.00	0.02 ± 0.00
Standard lotion (SL)	0.65 ± 0.02	0.86 ± 0.02	0.12 ± 0.21	3.67 ± 0.16
SL + KA (5%, *w*/*w*)	0.35 ± 0.02	0.30 ± 0.041	3.23 ± 0.44	2.20 ± 1.19
SL + KAD (5%, *w*/*w*)	0.69 ± 0.02	0.47 ± 0.02	3.03 ± 0.50	1.02 ± 0.10
CL_1_ Vaseline^®^	0.86 ± 0.02	0.33 ± 0.01	0.022 ± 0.00	0.29 ± 0.01
CL_2_ Safi^®^	0.35 ± 0.01	0.34 ± 0.01	2.29 ± 0.09	1.83 ± 0.20
CL_3_ Nivea^®^	0.39 ± 0.01	0.31 ± 0.03	0.65 ± 0.02	0.86 ± 0.21
